# Sortilin drives hypertension by modulating sphingolipid/ceramide homeostasis and by triggering oxidative stress

**DOI:** 10.1172/JCI156624

**Published:** 2022-02-01

**Authors:** Fahimeh Varzideh, Stanislovas S. Jankauskas, Urna Kansakar, Pasquale Mone, Jessica Gambardella, Gaetano Santulli

**Affiliations:** 1 Department of Medicine, Division of Cardiology, Wilf Family Cardiovascular Research Institute, Einstein–Mount Sinai Diabetes Research Center (ES-DRC), Fleischer Institute for Diabetes and Metabolism (FIDAM), and; 2 Department of Molecular Pharmacology, Institute for Aging Research, Institute for Neuroimmunology and Inflammation (INI), Albert Einstein College of Medicine, New York, New York, USA.

## Abstract

Sortilin is a glycoprotein mainly known for its role as a trafficking molecule directing proteins to specific secretory or endocytic compartments of the cell. Its actual contribution to essential hypertension has remained hitherto elusive. Combining top-notch in vivo, ex vivo, and in vitro approaches to clinical investigations, Di Pietro et al. explored the signaling pathway evoked by sortilin in endothelial cells and report on such exploration in this issue of the *JCI*. The researchers identified circulating sortilin as a biomarker associated with high blood pressure. Mechanistically, they demonstrate that sortilin altered sphingolipid/ceramide homeostasis, initiating a signaling cascade that, from sphingosine-1-phosphate (S1P), leads to the augmented production of reactive oxygen species. Herein, we discuss the main implications of these findings, and we anticipate some of the potential avenues of investigation prompted by this discovery, which could eventually lead to treatments for cardiometabolic disorders.

## What is sortilin?

Sortilin is an approximately 95 kDa single-pass type I transmembrane glycoprotein and a member of the vacuolar protein sorting 10 protein (VPS10P) domain receptor family, which also includes sortilin-related receptor 1 (SORL1, also known as sorting protein–related receptor containing LDLR class A repeats [SorLA]) and sortilin-related VPS10 domain containing receptor 1–3 (sorCS1-3) (1–3). The gene encoding for sortilin, *SORT1* (also known as neurotensin receptor 3 [NTR3] or glycoprotein 95 [Gp95]), resides on human chromosome 1 at the band 1p13.3 (whereas it is located on murine chromosome 3) and includes 23 exons. The protein is composed of a short cytoplasmic tail (carboxyterminal domain, involved in its internalization), a single transmembrane helix, and a large extracellular Vps10p domain (75 kDa) making up a cysteine-rich domain and a ten-bladed β propeller with an inner tunnel that contains multiple ligand-binding sites. Sortilin acts as a receptor, a coreceptor, and a trafficking molecule that binds and directs proteins to specific secretory or endocytic compartments of the cell and regulated pathways, including lysosomes, endosomes, and exosomes, or to the cell surface, where it also participates in the trafficking of extracellular vesicles (4–8). Less than one-tenth of the cellular sortilin pool is actually present at the cell surface, whereas it is mainly located in intracellular membrane structures involved in the sorting of various ligands.

## Sortilin and arterial hypertension

Initially identified in brain tissue and mostly studied in neurological disorders (4), sortilin has been later associated with a number of pathological conditions, including dyslipidemia, inflammation, insulin resistance, atherosclerosis, and vascular calcification (7, 9). In this issue of the *JCI*, Di Pietro and collaborators elegantly demonstrate that sortilin is also functionally linked to endothelial dysfunction and hypertension (10). The authors identified sortilin as a powerful biomarker associated with high blood pressure, since the increase in circulating sortilin levels (alongside sphingosine-1-phosphate [S1P] and soluble NADPH oxidase 2–derived [NOX2-derived] peptide) was more pronounced in uncontrolled hypertensive patients. This trailblazing discovery has obvious implications for the stratification and clinical management of hypertension.

## Sortilin drives oxidative stress and hypertension

**Figure 1:**
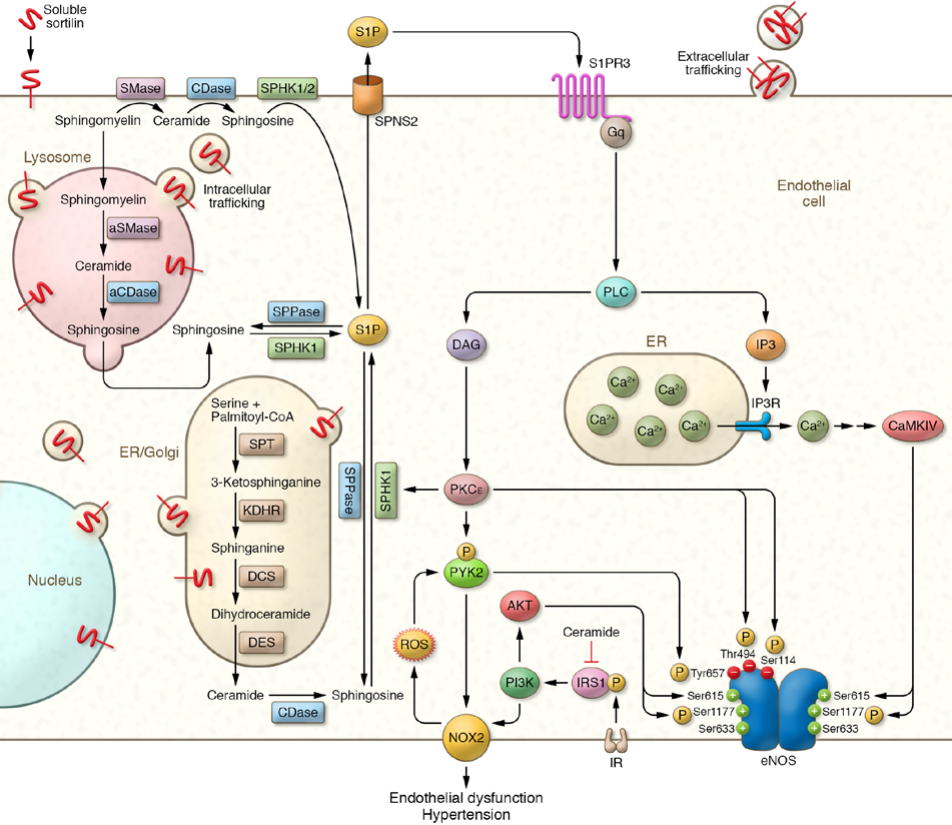
Main molecular pathways activated by sortilin and the sphingolipid/ceramide signaling cascade in the endothelial cell. Hypertensive patients display endothelial dysfunction, dysregulation of the sphingolipid/ceramide homeostasis, and elevated oxidative stress. Sortilin levels are linked to endothelial dysfunction and hypertension, and circulating sortilin is proposed as a biomarker associated with high blood pressure. Sortilin, by orchestrating the intracellular trafficking of enzymes implicated in the metabolism of ceramides, signals through the S1P pathway, augmenting ROS production and eventually impairing endothelium-dependent relaxation. Specifically, S1P triggers S1P3-mediated signaling, leading to NOX2 activation via PKCɛ and PYK2. Sortilin activated a precise pathway that is independent of specific eNOS phosphorylation sites, including Ser1177 and Thr494. The phosphorylation sites of eNOS can activate or inhibit the enzyme; the inhibitory sites are Ser114 (phosphorylated by PKCε), Thr494 (phosphorylated by PKCε; actually, the threonine is in position 492 in human eNOS, in position 494 in murine eNOS, and in position 495 in bovine eNOS), Tyr657 (phosphorylated by PYK2); the sites activating eNOS are Ser615 (phosphorylated by AKT and CaMKIV), Ser633 (phosphorylated by PKA, not included in the figure), and Ser1177 (phosphorylated by AKT and CaMKIV). aCDase, acid ceramidase; CaMKIV, Ca^2+^/calmodulin-dependent protein kinase IV; DAG, diacylglycerol; DCS, dihydroceramide synthase (also known as ceramide synthase 3 [CS3]); DES, dihydroceramide desaturase; ER, endoplasmic reticulum; Gq, G protein α subunit q; IP3, inositol 1,4,5-trisphosphate; IP3R, inositol 1,4,5-trisphosphate receptor; IR, insulin receptor; KDHR, 3-ketodihydrosphingosine reductase; NOX2, NADPH oxidase 2; PI3K, phosphatidylinositol 4,5-bisphosphate 3-kinase; SPHK, sphingosine kinase; SPNS2, sphingolipid transporter 2; SPPase, S1P phosphatase; SPT, serine palmitoyltransferase.

Starting from the well-known contribution of sortilin in lipid raft clustering and in the intracellular trafficking of acid sphingomyelinase (aSMase) (11), an enzyme responsible for the hydrolysis of sphingomyelin to ceramide and phosphocholine, the authors surmised a functional role for S1P in the regulation of oxidative stress in endothelial cells. Such a role was proven through means of a combination of in vivo, ex vivo, and in vitro models. Pharmacologic and genetic approaches revealed that S1P activated the type 3 S1P receptor (S1P3), initiating a signaling pathway (Figure 1) that includes PKCε and proline-rich tyrosine kinase 2 (PYK2, also known as protein tyrosine kinase 2 β [PTK2B]), leading to the generation of reactive oxygen species (ROS) by NOX2 (also known as cytochrome B-245 β chain, CYBB, or gp91-phox). It is interesting to note that both aSMase and neutral sphingomyelinases (nSMase) are redox-sensitive enzymes whose activities are incremented by ROS, thereby creating a harmful vicious cycle.

## Endothelial dysfunction and hypertension

A healthy endothelium promotes vasodilation (mostly attributable to NO, adenosine, and prostacyclin), has an atheroprotective action dependent on antioxidant and antiinflammatory effects (including inhibition of leukocyte adhesion and migration), has anticoagulant and profibrinolytic effects, and counteracts vascular permeability. On the flip side, a dysfunctional endothelium is characterized by impaired vasodilation and augmented vasoconstriction (mainly mediated by angiotensin II, endothelin-1, and thromboxane A2) and elevated proinflammatory and procoagulatory events as well as amplified permeability and increased oxidative stress (12–14).

Production of nitric oxide (NO), obtained in endothelial cells from its precursor l-arginine (15) via the enzymatic action of endothelial NO synthase (eNOS), is a hallmark of a properly functioning endothelium. However, the exact molecular mechanisms leading to reduced NO generation, as observed in hypertension, are not fully clear. The experimental evidence provided by Di Pietro et al. (10) combining clinical and preclinical data adds another key piece to the jigsaw puzzle of essential hypertension.

## Is there a vascular ceramide/S1P rheostat?

For years, sphingolipids have been considered mere intermediate products of sphingosine degradation until their complex effects began to be delineated. Indeed, in addition to being structural components of the eukaryotic membranes, sphingolipids act as signaling molecules that regulate a number of biological functions. In 1996, two interconvertible sphingolipid metabolites, namely, ceramide and S1P, were identified as major determinants of cell fate, a concept referred to as the “sphingolipid rheostat” (16), attempting to connect several independent findings evidencing the capacity of ceramide and S1P to differentially regulate cell survival and cell growth by modulating opposed signaling pathways. Increased ceramide levels induce cell growth arrest and apoptosis, while S1P suppresses ceramide-mediated apoptosis and is necessary for cell proliferation induced by growth factors. Later on, ceramide and S1P emerged as fundamental regulators of vascular tone (17) at the level of both endothelial and vascular smooth muscle cells (VSMCs).

S1P can act as a paracrine or autocrine mediator by binding to specific membrane receptors (divided into five subtypes: S1PR1–5) that orchestrate fundamental biological processes, including cell proliferation, migration, chemotaxis, cytoskeleton organization, angiogenesis, and mitogenesis. These receptors are also involved in immune modulation and in the suppression of innate immune responses from T cells. Therefore, the signaling pathways linking S1P3 and ROS generation could be implied in the two-way relationship between cardiovascular and immune systems, which might be involved in the pathophysiology of several cardiometabolic disorders, especially considering that sortilin regulates the secretion of proinflammatory cytokines from immune cells (8).

## S1P receptors and vascular tone

At nanomolar concentrations, S1P elicits endothelium-dependent vasodilatation (via NO), whereas at higher concentrations (1–100 μM), reached for instance in the presence of thrombi, S1P induces vasoconstriction. Intriguingly, S1P3s are crucial in S1P-mediated vasoconstriction, as shown in experiments conducted in cerebral arteries from rodents (18); thus, S1PR signaling in the endothelium could counteract S1PR vasoconstriction mediated by VSMCs.

On the other hand, ceramide has been implicated in endothelial oxidative stress, growth inhibition, alterations of the cytoskeleton, senescence, and apoptosis; nonetheless, its exact role in the regulation of vascular tone remains debated. Despite some reports that have shown that ceramide could enhance eNOS expression and phosphorylation, substantial evidence indicates that ceramide is able to increase ROS production (both mitochondrial and cellular ROS), reduce NO bioavailability, facilitate the interaction of eNOS with its negative regulator caveolin-1, and potentiate the phosphorylation of eNOS at negative regulatory sites.

To rule out alterations in eNOS phosphorylation in the signaling pathway activated by sortilin, Di Pietro and colleagues cunningly measured eNOS phosphorylation at the activation site Ser1177 as well as at the inhibition site Thr494, finding no notable differences between human endothelial cells stimulated with acetylcholine alone and cells pretreated with sortilin for one hour (10). However, other eNOS phosphorylation sites were not explored, including the inhibitory sites Ser114, which is targeted by PKCε, and Tyr657, which is targeted by PYK2 (Figure 1). Remarkably, increased phosphorylation at any of these sites would not have contradicted the findings shown in the paper, inasmuch as both PKCε and PYK2 are downstream of the signaling pathway that follows S1P3 activation by S1P, and PYK2 can activate NOX2 (leading to increased ROS production) and at the same time is further activated by ROS (Figure 1). Moreover, Joel Karliner’s team had previously shown in a murine model of myocardial ischemic preconditioning that sphingosine kinase is a target of PKCε (19).

## Sortilin and insulin resistance

The fil rouge linking endothelial dysfunction, hypertension, and diabetes is complex and not fully understood, and a primary role for sortilin in the common mechanisms underlying these processes cannot be excluded. Indeed, in addition to its action at the endothelial level, sortilin controls lipid absorption from the intestine and regulates glucose transporter type 4 (GLUT4) storage vesicles in skeletal muscle and adipocytes, which is significant because a compromised translocation of these vesicles is involved in the development of diabetes; furthermore, sortilin mRNA and protein are downregulated in adipose tissue and muscle from obese *ob/ob* and *db/db* mice, and insulin resistance has been shown to induce hepatic sortilin degradation. Intriguingly, in a murine model of diet-induced obesity (DIO), sortilin-deficient rodents exhibited enhanced glucose uptake in insulin tolerance tests and gained less body weight than wild-type mice (5). This favorable metabolic effect could be at least in part attributed to an altered aSMase activity, most likely reducing the levels of ceramide, which is a well-recognized negative modulator of insulin signaling, by inhibiting the insulin receptor substrate 1 (IRS1) (Figure 1). Of note, SORL1 has also been shown to promote insulin-induced suppression of lipolysis in adipocytes by acting as a sorting factor for the insulin receptor that redirects internalized receptor molecules from endosomes to the plasma membrane, thus enhancing the expression of the insulin receptor and strengthening insulin signal reception in target cells (9).

At the level of pancreatic islets, sortilin-derived peptides promote β cell survival via the CaMK/CREB signaling pathway (20); similarly, when associated with a neurotensin receptor, sortilin protects pancreatic β cells from stress-induced apoptosis. Instead, when associated with the p75 neurotrophin receptor (p75NTR), sortilin could favor β cell death.

## Future directions for sortilin interactomics and beyond

The novel findings summarized above will certainly open up broad research opportunities. Future endeavors could include identifying the molecular mechanisms leading to sortilin cleavage/shedding (most likely released by activated platelets) and determining whether changes in plasma levels of sortilin may have causative or consequential effects in arterial hypertension, studying the potential role of sortilin in endothelial autophagy (21), which might underlie the pathogenesis of hypertension and other cardiovascular diseases, and elucidating the involvement of endothelial sortilin in the pathophysiology of dyslipidemia, especially given the interaction between sortilin and PCSK9 (22).

Based on recent transcriptomic analyses in zebra fish showing that mutations in SORL1 affect mitochondrial function (23), further studies are warranted to determine whether sortilin could regulate mitochondrial fitness and mitochondrial ROS production. Last but not least, sortilin dimerization has been implied in the formation of extracellular vesicles, a heterogeneous group of cell-derived membranous structures comprising exosomes and microvesicles (Figure 1), which regulate intercellular communication by transferring microRNAs, proteins, and lipids to neighboring cells (24). Since endothelial extracellular vesicles have emerged as critical players in a number of cardiovascular and cerebrovascular disorders, including the systemic manifestations of COVID-19 (25), we anticipate that exciting research will stem from the just-established participation of sortilin in endothelial dysfunction.

## Author contributions

The order of co–first authors was determined by drawing lots.

## Version 1 02/01/2022

Electronic publication
